# Assessment and Improvement Strategies for a Breast Cancer Early Detection Program in Rural South Africa

**DOI:** 10.1200/JGO.18.00015

**Published:** 2018-06-21

**Authors:** Megan Hadley, Lisa A. Mullen, Lindsay Dickerson, Susan C. Harvey

**Affiliations:** **Megan Hadley** and **Lindsay Dickerson**, The Johns Hopkins University School of Medicine; and **Lisa A. Mullen** and **Susan C. Harvey**, Johns Hopkins Medicine, Baltimore, MD.

## Abstract

**Purpose:**

To assess and develop solutions for an ultrasound-based breast cancer early detection program in rural South Africa 1 year after implementation.

**Methods:**

A WHO-endorsed RAD-AID Radiology Readiness Assessment was used to evaluate clinic resources. In addition, 5 weeks of observation identified resource deficiencies and reviewed existing documentation methods. On the basis of stakeholders’ input and the BI-RADS, we developed new documentation systems. Training was followed by a survey that assessed feasibility and provider acceptance.

**Results:**

Resource limitations included lack of computers, unpredictable electrical supply, and inconsistent Internet. The assessment revealed incomplete documentation of breast clinical examinations and history, breast lesions, and follow-up. Furthermore, limitations negatively affected communication among providers. Three solutions were developed: a paper patient history form, a paper clinical findings form, and a computerized patient-tracking data base compliant with BI-RADS. Three nurses, three nursing assistants, and one counselor completed the survey. Seventy-one percent indicated positive general attitudes, and 100% agreed that the documentation system is easy and useful and improves overall quality of care, follow-up, decision making; access to clinical information; and communication between clinicians and patients. Five of the seven providers reported that the system increased visit time, but three of those five believed that the process was valuable.

**Conclusion:**

Implementation of a breast cancer early detection program in resource-limited regions is challenging, and continual assessment is essential. As a result of identified needs, we developed a documentation system that was broadly accepted. Future steps should focus on increasing efficiency, evaluation of provider attitudes long term, and clinical effect.

## INTRODUCTION

Breast cancer is a leading cause of cancer deaths in women globally and caused more than half a million deaths in 2011.^1-4^ Data reveal that more than one half of breast cancer deaths occur in low- and middle-income countries (LMICs), where survival rates are 60%, at best, compared with > 80% in North America for all stages.^1-3^ Definitive diagnosis occurs at later stages in LMICs, when treatment is less effective, and reflects inadequate screening for early detection, lack of education and awareness, and limited diagnostic methods.^1,2,5-7^ For example, in a study by Vorobiof et al,^8^ 78% of black women in South Africa presented at stages 3 and 4. Appropriate programs that improve detection, diagnosis, and treatment of breast cancer in LMICs are essential to improving outcomes.^9,10^

In 2016, we collaborated with Hlokomela Clinic, a community health organization in Hoedspruit, South Africa, that serves > 30,000 visits annually, with outreach clinics serving an additional 170,000 patients. The clinic was chosen because of its nonprofit status and long-standing positive presence in the community and because its patient population demographically represents African women who live in rural areas. The ability to access local diagnostic and treatment resources, reach a large number of women, and implement appropriate strategies by using the available ultrasound unit were vital considerations for establishing this program because these factors would greatly influence the program’s ability to have an effect on morbidity and mortality.^11^

The program aimed to take an initial step toward improving breast cancer outcomes by implementing earlier detection methods because the 5-year breast cancer survival rate in rural South Africa was reported to be only 53% between 2005 and 2009.^7,12^ Nurses, nurse assistants, and lay counselors were trained to perform clinical breast examination, educate patients on breast self-examination, and perform breast ultrasound as part of a potentially scalable breast cancer care program in one location in South Africa.^12^

In low-resource regions, mammography—the standard-of-care breast cancer screening tool in developed countries—is not always feasible. In these settings, clinical breast examination followed by breast ultrasound is an inexpensive and more widely accessible method for earlier breast cancer detection.^1,13^ Ultrasound may have comparable detection rates to that of mammography^14^ and is especially useful in women with symptoms that include palpable lumps or focal pain and in women with denser breast tissue.^15-17^ Studies of similar programs have demonstrated effective use of midlevel providers to perform ultrasounds in regions with low physician density.^18,19^ Our early detection program has shown initial success with applying these methods, and our focus is now on securing longer-term success.

Sustainability of such programs requires ongoing efforts and oversight, including quality assurance and maintenance of trained personnel proficiency.^13^ LaGrone et al^17^ advocated for regulation and follow-up of LMIC ultrasound programs, but a lack of literature remains with regard to follow-up to ensure long-term program success.

In addition, accurate and accessible clinical documentation is an essential component of health care delivery and communication among providers. A policy position paper from the American College of Physicians discussed the development of clinical documentation as a method of both tracking conditions longitudinally and communicating health care decisions to other care team members.^19^ When considering breast cancer care, the recording of risk factors and changes in symptoms or imaging over time is important. Therefore, adequate clinical documentation is an essential component of a successful breast cancer detection initiative.

Since the implementation of the Hlokomela Clinic program, trained providers have used their knowledge to evaluate three to five women each day and have reached 500 in 10 months with a combination of clinical breast examination and breast ultrasound. A practicing breast radiologist from Helen Joseph Hospital, part of University of Witwatersrand (Johannesburg, South Africa), provided several onsite and hospital-based follow-up trainings for providers. Despite maintaining provider proficiency, there remained an insufficient understanding of what additional program challenges existed. This study aimed to understand what needs evolved in the year after implementation of the early detection program and to evaluate solutions, with an area of particular interest in quality of clinical documentation.

## METHODS

A multimodal needs assessment was performed to review the recently implemented breast cancer early detection program at the Hlokomela Clinic and consisted of the WHO-endorsed RAD-AID Radiology Readiness Assessment for evaluating radiology and clinic infrastructure as well as 5 weeks of observation. The 16-section readiness assessment (Data Supplement) was used to investigate multiple aspects of the clinic’s infrastructure, including human resources, structural features, and communication methods, designed for the development of sustainable solutions to radiologic and clinic needs on the basis of both what exists and what is absent.

Observation included an initial week of rapport building with clinic staff by a medical student under the guidance of a practicing breast radiologist, which allowed for sharing of clinic values before data collection and integration of the researcher into the clinical routine. Next, 4 weeks of focused observation that emphasized workflow patterns, standard practice, and setting-specific characteristics and limitations were documented in daily field notes.^20^

We also focused on a review of the existing clinical documentation system by using the American College of Radiology Practice Parameters for Communication of Diagnostic Image Findings, the Breast Imaging Reporting and Data System (BI-RADS), the Breast Cancer Surveillance Consortium report on data for evaluating screening performance in practice, and patient documentation used in the breast imaging division of Chris Hani Baragwanath Hospital (Johannesburg, South Africa) for comparison.^21,22^ The methods for the new documentation system were based on Kaplan’s social interaction framework of information technology development, which incorporates end-user input.^23,24^ Stakeholders were directly involved in design and feedback to pair methods and materials with the needs and resources of the clinic accurately. Through this strategy, three documentation tools were developed: a patient history and risk assessment paper form, a clinical assessment paper form, and an electronic patient-tracking data base. Two breast radiologists previously involved in program implementation and provider training evaluated the documents for adequate content. The documentation tools were then trialed at Hlokomela Clinic for 1 week.

Stakeholder feedback was obtained and the documents modified to improve clarity, reduce errors, and limit complexities. Staff training on document use combined previously described medical education models of demonstrations, peer teaching, and supervised clinical experience.^25^ In addition, a new reliable and reproducible method for storage of the breast-specific patient records was implemented. Providers were educated on where and how to store data collected within the patient charts.

After full implementation, participants were asked to complete a confidential survey evaluation of the documentation system, which was designed to gauge feasibility, provider attitudes, and early effect. The survey included 16 scaled and three open-ended responses. Scaled responses used a five-point Likert scale that ranged from strongly agree to strongly disagree.

## RESULTS

Data gathered from the needs assessment indicated several key areas for quality improvement within the program: a complete and consistent method of documenting patient visits; improvement in tracking and documenting patient follow-up and referral; continuing education and skill building opportunities for staff members; and an image archiving and communications system for viewing images, tracking lesions, and facilitating referrals and interprovider communication.

### Documentation of Patient Visits

Early observation of patient documentation highlighted several components that did not meet the BI-RADS and Breast Cancer Surveillance Consortium recommendations. Namely, breast ultrasound documentation should include indication for the examination, statement of scope and technique, description of breast composition, clear description of relevant findings, comparison with previous examinations, assessment, and management.^20,21^ Documentation at the start of the study was missing several of these categories, and the ones included were not consistently completed. Resource limitations observed and considered relevant to documentation procedures included limited availability of computer workstations for providers, an unpredictable power supply, and use of paper rather than electronic charting and documentation systems.

### Documentation of Patient Follow-Up

**Fig 1:**
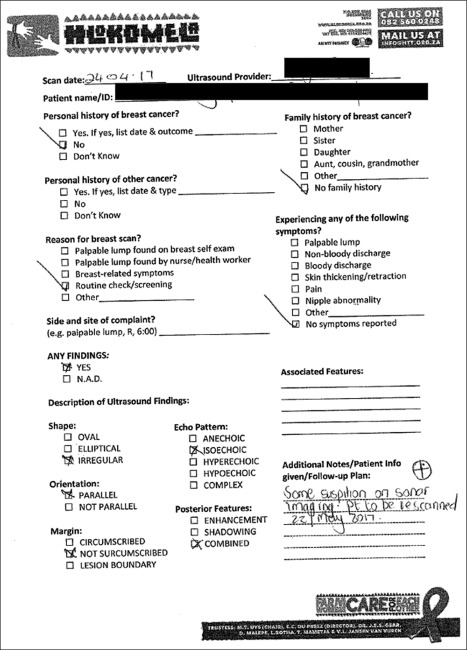
An example of a completed standard documentation form before the study. Several Breast Imaging Reporting and Data System criteria are not included, and multiple sections are not fully completed. N.A.D., no appreciable disease.

Although a referral and follow-up system existed, a lack of clear documentation of the recommended next steps in care existed. When a follow-up plan was indicated, it was filed in the patient’s paper chart chronologically and mixed among other unrelated clinical documents where clinic staff was unlikely to see it when the patient returned. No system was present for tracking lesions over time or determining completion of follow-up care. Figure 1 shows an example of a completed standard documentation form before the study. When a patient was referred to another clinic for treatment, no documentation was kept on the patient’s care or outcome at the outside institution. The documentation loop was not closed as required by BI-RADS.

### Continuing Education

Results of the RAD-AID survey indicated that online training, print journals, online journals, local meetings, and international meetings were rarely or never accessible to sonographers for continuing education. In-person training was listed as available but in limited supply. Some comments stated that many available trainings are not financially accessible.

### Image Storage

Observation of standard clinical practice revealed storage of ultrasound images directly on the ultrasound machine. The images would then be printed on paper for use by providers at the referral clinic. Our assessment indicated the following key challenges to image storage and viewing: data transmission available but with occasional interruptions, lack of general workstations for all staff, no electronic medical record system, no radiology information system, no teleradiology, no digital radiology viewing workstations, and no picture archiving and communication system. Although these standards are based on higher-income communities, we used them in our assessment to determine a starting point for intermediate goals.

**Fig 2:**
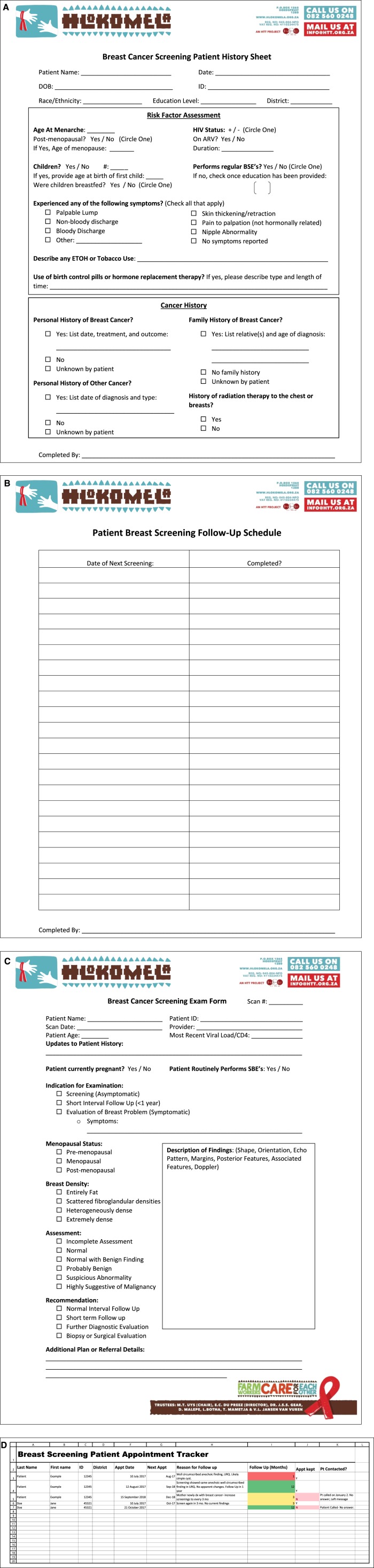
New documentation system components, including (A) a patient history and risk assessment paper form, (B) screening follow-up schedule paper form, (C) a clinical assessment paper form, and (D) an electronic patient-tracking database. ARV, antiretroviral; BSE, breast self-examination; DOB, date of birth; ETOH, ethanol; SBE, self-breast examination.

On the basis of the needs assessment results, we developed solutions for two of the needs we deemed to be most critical to patient care and solvable within the constraints of setting-specific limitations: documentation of patient visits and documentation of follow-up. Because of limited computer access and provider time constraints, we developed a paper form for documentation of the patient visit, including patient information, cancer history, family history, indication for the examination, ultrasound findings, and follow-up plan (Fig 2).

We incorporated a structured system that indicates to all clinic providers when a patient is due for follow-up breast care. We designated a consistent location in the patient chart for breast examination documentation so that clinical and ultrasound findings in subsequent visits could be compared easily. In addition, we developed an electronic system for monthly use, which organized patients by follow-up date, reason for follow-up, and contact information. Follow-up recommendations and subsequent care were documented in the computer database.

After implementation of the documentation system, feedback surveys were completed by all seven staff members trained on document use, including three nurses, three nursing assistants, and one counselor, all of whom were either directly involved in breast screening or referral of patients to the women’s clinic. In rating overall attitudes toward the documentation system, 71% (five of seven) indicated positive or very positive general attitudes, with the remaining 29% indicating neutral attitudes (none negative); 100% of these staff members agreed or strongly agreed that the clinical documentation system is useful and easy to use and improves overall quality of care, patient follow-up, clinical decision making, access to clinical information, communication among clinicians, and communication between clinicians and patients. In an assessment of clinical burden, five of the seven staff members agreed that the documentation system increased length of patient visits, but three of these five found the amount of time spent on documentation worthwhile.

## DISCUSSION

Programs for early detection of breast cancer in LMICs have immense potential to reduce global morbidity and mortality by identifying stages when treatment is effective in prolonging or saving lives. The initial implementation phase of a breast cancer early detection program is only the first step in developing a successful, locally sustainable program. As our assessment demonstrated, continued support, follow-up, and consideration of program needs are essential for quality improvement and sustaining these programs. One of the common and critical areas for a high-value program in any medical setting is appropriate documentation. In LMICs, documentation design should take into consideration setting-specific characteristics and limitations.

Although several areas of the program were identified for improvement, we chose to focus our efforts on understanding and remedying patient visit documentation, with a specific focus on the clinical breast examination and breast ultrasound. Factors that influenced this decision were maximizing the effect on patient care and outcomes, time and resource limitations, and our perceived ability to make critical improvements rapidly. We believed that improvements in documentation would be a catalyst for progress in other realms.

Evaluation of available resources guided the development of an appropriate documentation system that was easily adopted and widely accepted by providers. With unreliable Internet and few computer workstations, paper charting was the most feasible solution for documentation and prevented an extension of visit times while providers queued for computer availability. Early feedback from the staff influenced the formatting, including use of a checkbox format that offered quick and accurate recording of data to facilitate the eventual move toward standardized structured reporting. Both resource matching and integration of stakeholder input improved overall enthusiasm and acceptance of the new forms among providers.

By taking into account user feedback that checkboxes are fast and easy to complete and that space is needed for description of imaging findings, the existing forms were updated. The patient history form was expanded and separated from the clinical examination and imaging form, which permitted inclusion of additional risk factors (Fig 2A) and prompted providers to ask pertinent questions consistently and to record answers on an organized and rapidly completed form. The new clinical examination and ultrasound form (Fig 2C) was based on BI-RADS and included indication, assessment, and recommendations in checkbox format.

The description of ultrasound findings remained in free-text format, with space for lesion descriptors even in the event of multiple breast findings. We provided a list of descriptors at the top of the free-text area to prompt providers to be consistent with BI-RADS terminology (Fig 2C). In addition, the document training education included refresher training on BI-RADS lexicon for assessing findings. Providers were given access to supplemental lexicon education material for reference when completing forms (Data Supplement). The repetitive review of BI-RADS lexicon provided staff with an accurate and consistent vocabulary for the description of findings and reinforced the selection of the correct final assessment. In turn, the BI-RADS assessment category is directly linked to clinical recommendations and, therefore, patient management. We believe that these practice patterns will improve consistency and care over time. With this goal in mind, our next step is to move to a uniform checkbox ultrasound lexicon and ultimately to implementing this electronically after funding for technology is in place.

We also focused on improving patient follow-up documentation. By leveraging the existing HIV clinic care model of requiring monthly patient visits for medication adherence, we organized a single location in the patient chart where the next breast examination date was indicated, which gave providers the ability to monitor patient follow-up schedules at each visit. Furthermore, the electronic tracking system provided a method of rapid retrieval of patients lost to follow-up each month who could then be contacted.

In response to the documentation revisions, clinic providers acknowledged that this system that is based on the BI-RADS structure enhanced their ability to communicate and make clinical decisions, which may play a role in improving outcomes for the early detection program. One hundred percent of comments about the documentation system were positive. One provider commented, “I enjoy the time spent going through every question with my patient. This helps to have a holistic approach. Makes the visit and info gained thorough.”

Another anticipated, but not yet assessed benefit is reduction in unnecessary repeat imaging by clearly documenting when and what was already imaged. One provider stated, “It makes [it] easy to follow-up patients. Also to follow results after the scan.” Another mentioned that the new system “makes tracking easy and allows for more control over follow-ups.” In addition, this documentation system set up a method of data collection that can be used for more quality improvement and outcomes assessments.

Limitations of this project include an inability to address all needs identified by the assessment and in-person observation. As a result, we prioritized the needs and chose the solution best aligned with the available resources, patient care needs, and staff acceptance.

Future steps for the documentation project include evaluation of long-term provider attitudes because stakeholder motivation may help to guide and predict success of the program.^26^ We also hope to assess the system’s effect on patient outcomes, including appropriate patient follow-up care and, importantly, the ability to move forward to more electronic medical record keeping.

Additional future plans for the clinic are to address the other needs identified, including image storage, continuing education for providers, and a structured quality review program for both imaging and performance metrics. We plan to do this by collaborating with RAD-AID through access to its extensive volunteer team as well as its expertise in resource-appropriate image archive systems. The continuing education work has been partially addressed by providers who attended breast ultrasound training courses in Johannesburg, which we hope will continue through teaching files and RAD-AID education programs. Referral patterns continue to strengthen in the region as the surrounding district hospitals learn more about the program and partner within the regional health network.

In conclusion, implementation of a program for early detection of breast cancer in resource-limited regions is challenging. Our needs assessment revealed limited resources and suboptimal clinical operations that affected patient care and provider communication. These findings demonstrate that continued assessment of evolving needs and program workflow is essential for long-term success and sustainability. To our knowledge, the study provides novel information on methods for program evaluation after implementation. We determined that improvement of clinical documentation could have the greatest effect on initial follow-up. Thus, we implemented a new, relevant documentation system that the providers broadly accepted and that lays the foundation for future quality improvement initiatives. This assessment process and solution implementation may provide a framework for breast care documentation use and scaling in other resource-constrained settings.
